# Dynamic changes of brain functional states during surgical skill acquisition

**DOI:** 10.1371/journal.pone.0204836

**Published:** 2018-10-31

**Authors:** Somayeh B. Shafiei, Ahmed Aly Hussein, Khurshid A. Guru

**Affiliations:** 1 Department of Mechanical and Aerospace Engineering, University at Buffalo, SUNY, Buffalo, New York, United States of America; 2 Applied Technology Laboratory for Advanced Surgery (ATLAS), Roswell Park Cancer Institute, Buffalo, New York, United States of America; 3 Department of Urology, Roswell Park Cancer Institute, Buffalo, New York, United States of America; 4 Department of Urology, Cairo University, Cairo, Egypt; North Carolina Neuropsychiatry Clinics, UNITED STATES

## Abstract

There is lack of a standardized measure of technical proficiency and skill acquisition for robot-assisted surgery (RAS). Learning surgical skills, in addition to the interaction with the machine and the new surgical environment adds to the complexity of the learning process. Moreover, evaluation of surgeon performance in operating room is required to optimize patient safety. In this study, we investigated the dynamic changes of RAS trainee’s brain functional states by practice. We also developed brain functional state measurements to find the relationship between RAS skill acquisition (especially human-machine interaction skills) and reconfiguration of brain functional states. This relationship may help in providing trainees with helpful, structured feedback regarding skills requiring improvement and will help in tailoring training activities.

## Introduction

With the widespread adoption of robot-assisted surgery (RAS), it becomes crucial to provide robots with the capability of collaborating closely with humans in a complex and critical environment like the operating room (OR) [1, 2]. Understanding how learning and skill acquisition occur can open new horizons for surgical teaching, skill assessment, and also set a platform for active interaction with robots. This shared environment encompasses trainees acquiring the appropriate knowledge, learning the necessary surgical skills and how to interact with robots on one side, and robots should be equipped appropriately to learn new collaborative tasks on the other [3, 4].

The main challenge in skill assessment is the dynamic nature of learning, which results in brain neuroplasticity changes, where the brain’s neural organization changes during the development of a motor cognition skill through practice [5]. Learning technical surgical skills matures through 3 stages. First, the “cognitive” stage as one initially learns the skill and–thoughtfully- performs it. With practice, trainees become less thoughtful about the steps and reach “the associative stage” and can operate with fewer disruptions. The final stage is the “autonomous” stage, where the trainee can perform automatically without putting much thought, meanwhile paying more attention towards other aspects of surgery [1, 6, 7].

Several key brain functional features have been proposed for evaluating surgical skills proficiency, including kinematics and motor control. Hand movement kinematics has been the main source of information in various motor skill assessment studies [8–12]. Completion time, distance by hand, total distance traveled, speed, curvature, and relative phase of two hands trajectories as well as tool-based metrics of tool out of view, tools collision, tissue damage, camera control, number of critical errors, and clutch usage have been proposed for RAS skill evaluation [13–15]. However, tool-based metrics evaluate surgical skills based on static measurements and do not represent any dynamic effect and are not able to assess the associated change in brain functioning and brain neuroplasticity. Still, there is controversy about the optimal method for cognitive analysis during acquisition of RAS skills [6, 16–19]. Recording brain neural oscillations of surgeons while interacting with robots, utilizing electroencephalogram (EEG) neurofeedback, has been proposed to analyze functional dynamics of the brain of surgeons working in laparoscopy compared to those working in RAS [19]. EEG cognitive metrics, such as mental workload and level of engagement, have been used to analyze brain activity improvement during RAS learning [11, 19, 20]. However, skill-specific structured feedback to trainees could not be provided. It has been shown that modular training, defined as deconstruction of a procedure into smaller modules, can transfer technically challenging surgical skills in a step-wise fashion under appropriate supervision. Also, a structured feedback would be more constructive, as it allows tailoring of training activities and focusing on individual steps [21, 22].

High performance level in multifaceted tasks depends upon several factors including the learner’s ability to develop perceptual, cognitive, and motor skills [5]. The human brain is a complex system that includes various subsystems which interact with each other and dynamically change over different temporal scales while interacting with changes in the environment. It has been shown that during motor-cognition skill acquisition, there is a change of functional connectivity throughout areas of the brain [23, 24].

In this study, we hypothesized that the brain functional states reconfiguration during RAS skill acquisition can be informative toward monitoring surgeon’s performance progress during training. We analyzed the features extracted from brain functional network during RAS skill acquisition. Additionally, changes of brain states were quantified using dynamic network analysis to develop metrics for evaluation of performance in RAS. The main goal was to investigate dynamic changes of the brain functional states, and to find whether these changes are associated with performance improvement and practice time.

EEG has been previously used in different applications including mental workload measurement [25] gesture classification in computer aided design areas [26], and stress evaluation [18]. Here, EEG data were used to extract the brain functional network of RAS surgeon involved in different surgical tasks.

## Results

### Architecture of learning

**Fig 1:**
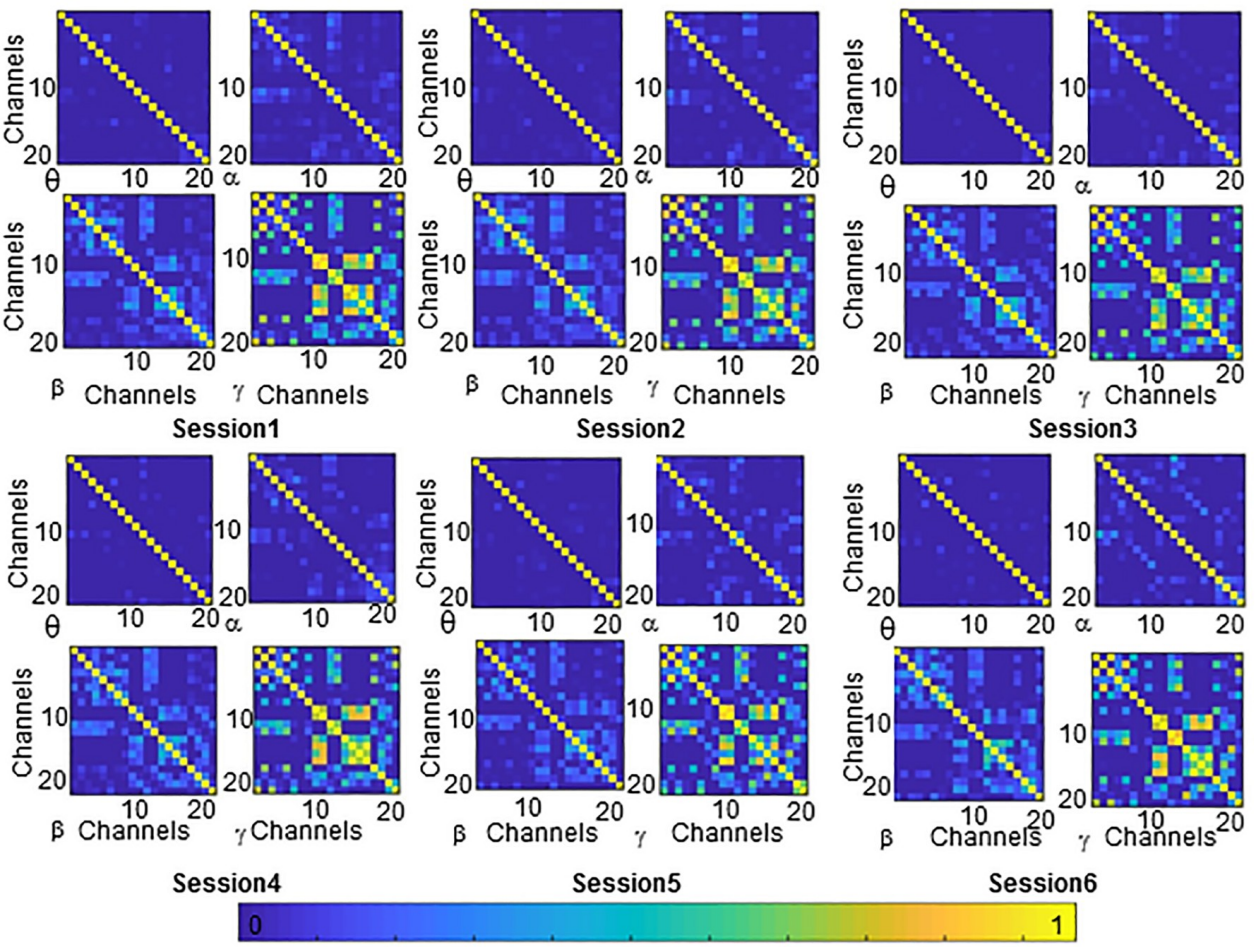
Architecture of RAS learning during 6 recording sessions, through frequency bands of θ, α, β, and γ channels 1–20 represent the EEG leads numbers. Channels 1–8 (motor area), channels 9–10 (Visual cortex), channels 11–16 (cognitive area), and channels 17–20 (other areas such as temporal cortex).

We first sought to address the question: “Are there sets of RAS surgeon’s brain areas that preferentially interact with one another during RAS tasks practice and learning?” To answer this question, we examined the behavior of a module allegiance matrix (MAM) throughout learning (sessions). Values of elements of this matrix (MAM_ij_) indicate the probability that two areas (channels i and j) be assigned to the same community, in the set of functional brain networks constructed from all subjects and recordings. MAM matrices during six sessions and different frequency bands were represented in Fig 1 (Data available in S1 File). These architectures display that channels in motor area (Frontal and Central channels [27]) consistently were assigned to the same community (recruited). The same thing happened to channels in cognitive (Prefrontal and Parietal channels [27]) and visual areas (Occipital channels [27]). This may suggest that areas relevant to task execution (motor and visual) and cognitive control (cognitive) are recruited consistently while other areas might only be transiently recruited.

### Dynamic architecture of learning

We then sought to address the question: “Does functional contribution of these consistent communities or their interactions change with practice during RAS learning process?” To answer this question, we used Integration and recruitment quantities (see Methods). All subjects started study without any RAS or human-robot interaction experience. During the study, we considered practice time (seconds) from session to session as practice time or experience of subject for the next session. It should be noted that practice time may not be the only effective variable here, as gap between practice sessions may also affect learning.

We used correlation analysis to be able to detect significant effects of practice time on integration and recruitment quantities and also investigate relationship between these quantities and performance level. Results of this investigation were represented in Table 1 (Data available in S2 File).

**Table 1:** Relationship between integration and recruitment through brain areas at different frequency ranges, and average performance level and practice time for recordings used for extraction of each module allegiance matrix through subjects and sessions. All 524 recordings during 6 sessions were considered to extract integration and recruitment values. Significant correlations (correlation >0.2 and P-value<0.05) are bolded.

	Recruitment (P-value)			Integration (P-value)			Frequency
	Motor	Cognitive	Visual	Motor-Visual	Motor-Cognitive	Visual-Cognitive	
**Performance Level**	0.56(0.24)	0.32(0.52)	-0.48(0.32)	0.61(0.19)	-0.58(0.22)	-0.66(0.15)	θ
	-0.17(0.73)	-0.36(0.48)	-0.31(0.53)	0.28(0.57)	-0.47(0.34)	-0.66(0.15)	α
	0.18(0.73)	0.12(0.81)	-0.43(0.38)	0.57(0.22)	**-0.84(0.03)**	-0.66(0.15)	β
	0.28(0.59)	0.53(0.27)	-0.16(0.76)	0.53(0.27)	-0.56(0.24)	-0.66(0.15)	γ
**Practice time (second)**	-0.17(0.74)	-0.20(0.70)	-0.72(0.10)	-0.79(0.05)	**-0.90(0.01)**	**-0.89(0.01)**	θ
	-0.72(0.10)	-0.22(0.68)	-0.41(0.42)	0.79(0.06)	-0.77(0.06)	**-0.89(0.01)**	α
	-0.45 (0.36)	-0.55 (0.25)	-0.73(0.09)	0.59(0.21)	-0.68(0.13)	**-0.89(0.01)**	β
	-0.30(0.55)	0.34(0.50)	-0.46(0.35)	**0.85(0.03)**	-0.81(0.05)	**-0.89(0.01)**	γ

Significant correlation between performance level and motor-cognitive integration during the β frequency band may indicate at higher performance levels (expertise improvement) tendency of channels in motor and cognitive modules to be integrated together decreases. It may suggest that autonomy of motor and cognitive modules increases to process information independently from each other, during β frequency band, by performance improvement. Beta waves are characteristics of a strongly engaged mind, able to perform complex mental processing [28]. Hence, improvement of motor and cognitive autonomy at βfrequency band (while brain is strongly engaged), by performance, may be an informative metric for performance evaluation.

Significant correlation between practice time and motor-cognitive integration during θ frequency band (unconscious band [28]), and motor-visual integration during γ frequency band (crucial frequency range for self-awareness and insight, cognition, and coordinating simultaneous processing throughout brain areas [28]), and visual-cognitive integration during all frequency bands indicates that autonomy of these modules increases by practice. These features may also be a good measurement for performance evaluation.

### Dynamic architecture after long gap in learning

We also sought to address the question: “Are the change in brain functioning permanent or does it deteriorate again?” Since our recording sessions were not regular and were limited to 6 sessions and also gap between practices was not equal for all sessions, to answer this question, we found the correlation between practice gap and integration and recruitment, represented in Table 2 (Data available in S2 File).

**Table 2:** Relationship between integration and recruitment of brain areas and average practice gap for recordings which were used for ‘module allegiance matrix’ extraction. All 524 recordings during 6 sessions were considered to extract integration and recruitment values. Significant correlations (correlation >0.2 and P-value<0.05) are bolded.

	Recruitment (P-value)			Integration (P-value)			Frequency
	Motor	Cognitive	Visual	Motor-Visual	Motor-Cognitive	Visual-Cognitive	
**Practice Gap**	-0.53(0.28)	-0.16 (0.78)	-0.43(0.39)	0.59(0.20)	-0.76(0.07)	-0.72(0.10)	θ
	**-0.81(0.04)**	0.39 (0.43)	-0.07(0.89)	0.75(0.08)	-0.65(0.15)	-0.72(0.10)	α
	**-0.87 (0.02)**	-0.65 (0.15)	**-0.85(0.02)**	0.2(0.70)	-0.39(0.44)	-0.72(0.10)	β
	-0.78(0.06)	0.03(0.94)	-0.06(0.90)	0.76(0.07)	-0.44(0.38)	-0.72(0.10)	γ

This result showed that a higher practice gap was associated with lower recruitment of motor (α and β), and visual (β) modules. This results in longer learning process. Although architecture of RAS learning seems to be permanent (Fig 1), dynamic architecture of the brain during learning was not permanent and it was affected by gap in practice. However, it should be noted that more recording sessions both before gap and after gap are needed to be able to answer this question more accurately, and to quantify the rate of deterioration and recovery.

### Change of RAS trainee’s brain functional network and cognitive features by task outcome (FSRS metrics) improvement throgh individual subjects and tasks

After investigating the dynamic architecture of learning, we also sought to address the question: “How does cognitive and functional connectivity features change by performance improvement during RAS skill acquisition?” To answer this question, we calculated the correlations between Fundamental Skills of Robotic Surgery (FSRS)metrics and cognitive and functional connectivity features through all subjects and recordings (Table 3; Data available in S3 File).

**Table 3:** Relationship between FSRS metrics, CT, and extracted EEG features (cognition and network) at different frequency bands. Twenty-seven subjects performed 5 tasks on robot simulator during six sessions, number of total recording was 524. However, Robotic Surgery Simulator (Ross) scores were not reported for some recordings. Total number of recordings considered in this correlation analysis was 260. Significant correlations (correlation >0.2 and P-value<0.05) are bolded.

	Clutch Usage (P-value)	Left Tool Grasp (P-value)	Left-Tool out of view (P-value)	# of-Errors (P-value)	Right-Tool Grasp (P-value)	Right-Tool Out-of-View (P-value)	Tissue Damage (P-value)	Tool-Tool Collision (P-value)
**CT**	**0.54** **(3.35x10**^**-21**^**)**	**0.41** **(5.03x10**^**-12**^**)**	**0.32** **(1.76x10**^**-7**^**)**	**0.39** **(2.7x10**^**-11**^**)**	**0.47** **(7.79x10**^**-16**^**)**	**0.47** **(9.8x10**^**-16**^**)**	**0.41** **(3.72x10**^**-12**^**)**	0.06 (0.03)
**Distraction**	0.16 (0.01)	-0.02 (0.79)	0.07 (0.28)	0.01 (0.91)	-0.03 (0.65)	0.04 (0.48)	0.07 (0.27)	0.03 (0.65)
**LE**	0.11 (0.07)	0.11 (0.07)	0.13 (0.04)	0.02 (0.73)	0.15 (0.01)	0.11 (0.06)	0.08 (0.22)	-0.02 (0.76)
**HE**	-0.10 (0.11)	-0.10 (0.13)	-0.12 (0.05)	-0.03 (0.66)	-0.12 (0.04)	-0.08 (0.20)	-0.08 (0.17)	0.02 (0.74)
**MW**	-0.15 (0.01)	0.003 (0.95)	-0.09 (0.17)	-0.10 (0.12)	-0.14 (0.02)	-0.07 (0.28)	-0.10 (0.13)	-0.08 (0.21)
**APA**	-0.13 (0.03)	-0.10 (0.1)	-0.07 (0.26)	-0.03 (0.64)	-0.17 (0.007)	-0.11 (0.07)	-0.02 (0.80)	0.02 (0.78)
**AI**	-0.01 (0.92)	-0.01 (0.92)	0.01 (0.82)	-0.20 (0.00)	-0.04 (0.49)	0.05 (0.38)	-0.05 (0.38)	-0.01 (0.84)
**Strength θ**	-0.18 (0.003)	-0.08 (0.21)	-0.11 (0.09)	-0.04 (0.51)	-0.18 (0.003)	-0.13 (0.03)	-0.11 (0.07)	0.00 (0.97)
**Strength α**	-0.19 (0.001)	-0.11 (0.08)	-0.13 (0.03)	-0.07 (0.26)	**-0.22** **(4x10**^**-4**^**)**	-0.14 (0.02)	-0.13 (0.03)	-0.02 (0.75)
**Strength β**	**-0.29** **(2.5x10**^**-6**^**)**	-0.15 (0.01)	-0.18 (0.004)	-0.15 (0.02)	**-0.25** **(4.9x10**^**-5**^**)**	**-0.23** **(0.002)**	-0.14 (0.02)	-0.03 (0.60)
**Strength γ**	**-0.33** **(3.6x10**^**-8**^**)**	-0.14 (0.02)	-0.17 (0.007)	-0.15 (0.01)	**-0.24** **(1x10**^**-4**^**)**	**-0.22** **(3x10**^**-4**^**)**	-0.11 (0.07)	-0.04 (0.55)
**Communication θ**	-0.18 (0.003)	-0.10 (0.10)	-0.10 (0.10)	-0.02 (0.71)	-0.18 (4x10^-3^)	-0.13 (0.03)	-0.10 (0.1)	0.03 (0.59)
**Communication α**	-0.18 (0.003)	-0.11 (0.07)	-0.11 (0.08)	-0.04 (0.51)	-0.19 (1x10^-3^)	-0.13 (0.04)	-0.10 (0.09)	0.03 (0.60)
**Communication β**	**-0.24** **(8.2x10**^**-5**^**)**	-0.14 (0.02)	-0.13 (0.04)	-0.12 (0.05)	**-0.22** **(3x10**^**-4**^**)**	-0.18 (0.003)	-0.11 (0.09)	0.00 (0.94)
**Communication γ**	**-0.29** **(2.7x10**^**-6**^**)**	-0.14 (0.02)	-0.12 (0.05)	-0.16 (0.01)	**-0.24** **(1x10**^**-4**^**)**	-0.18 (0.003)	-0.11 (0.08)	-0.01 (0.88)

Positive correlation between CT and most FSRS metrics is in agreement with results in the literature [20] because higher FSRS scores are associated with lower performance level and higher CT indicates lower expertise level and performance, too. Also, we detected significant negative correlations between network features (strength and communication) and FSRS metrics, which may suggest strength and communication as informative features for performance evaluation during RAS learning.

### Relationship between difficulty level, complexity level, practice time, and cognitive and brain network features and FSRS metrics

Although all designed tasks were considered for RAS learning, the complexity level (evaluated by expert surgeons through FSRS curriculum) of tasks were different. Also, difficulty level (evaluated by subjects not necessarily expert) calculated by using NASA-TLX scores showed that subjects evaluate tasks very differently. This likely stems from their ability (e.g. intelligence) as subjects are different. We then sought to address the question: “Do difficulty level and complexity level affect the outcome of subjects while performing tasks?” To answer this question, we calculated the correlation between these quantities and FSRS metrics (Table 4; Data available in S3 File). Results showed that performance level decreased (FSRS metrics increased) by increase of difficulty level. Our previous studies showed that NASA-TLX scores given by trainee are not reliable for expertise level assessment as we could not find any significant correlation between individual NASA-TLX metrics and expertise level (assessed by expert RAS surgeons) [20, 29], maybe because trainee doesn’t have enough proficiency to consider all necessary factors in evaluation [6, 20, 29].

**Table 4:** Correlations between task difficulty level, complexity level, and FSRS features at different frequency bands. Twenty-seven subjects performed 5 tasks on RoSS simulator during six sessions, number of total recording was 524. However, Ross scores are not reported for some recordings. Total number of recordings considered in this correlation analysis was 260. Significant correlations (correlation>0.2 and P-value<0.05) were bolded.

	Difficulty level (P-value)	Complexity level (P-value)
**Clutch usage**	**0.20 (1x10**^**-3**^**)**	0.003 (0.96)
**Left Tool Grasp**	**0.34 (1.03x10**^**-8**^**)**	-0.005 (0.93)
**Left-Tool out of view**	0.19 (2x10^-3^)	0.02 (0.74)
**# of Errors**	**0.38 (1.31x10**^**-10**^**)**	**0.43 (4.47x10**^**-13**^**)**
**Right-Tool Grasp**	**0.33 (5.74x10**^**-8**^**)**	0.09 (0.14)
**Right-Tool Out-of-View**	**0.27 (6.74x10**^**-6**^**)**	0.05 (0.41)
**Tissue Damage**	**0.22 (3x10**^**-4**^**)**	0.14 (0.02)
**Tool-Tool Collision**	0.04 (0.49)	-0.08 (0.17)

The results in Table 4 may suggest that although scores given to individual NASA-TLX are not reliable for expertise level assessment [6, 20, 29], difficulty level feature (calculated by using some of these metrics) can be an informative feature toward performance evaluation during RAS skill acquisition.

## Discussion

In this study we addressed the hypothesis that continuous practice during human-robot interaction (RAS skill acquisition) results in reconfiguration of brain functional states. We recorded EEG data from 27 subjects when performing four RAS surgical tasks, selected from FSRS curriculum focusing on human-machine interface skills and one advanced RAS task, on a simulator over one year. We used network analysis algorithms to extract functional states and investigate the change of RAS trainees’ brain network dynamics at different frequency ranges. We found that motor, cognitive, and visual areas formed three separate, functionally cohesive modules whose recruitment did not change by practice but whose integration changed at different frequency bands as performance became more automatic.

Since learning RAS skills is very complicated and improvement of performance and rate of learning depends on several factors including subject intelligence (we didn’t include this factor in our study), higher practice time may not be necessarily associated with higher performance level. Therefore, we investigated change of integration between motor, cognitive, and visual modules by performance improvement and practice time, separately. We observed that motor-cognitive intergration at βband decreased by performance improvement, while motor-visual and also cognitive-visual modules did not display significant changes by performance improvement. This result may introduce the motor-cognitive integration level at βfrequency band as informative feature to objectify RAS performance evaluation and also to determine the rate of learning RAS skills for individual subjects (trainees).

However, integration between all three modules showed significant changes, at different frequency bands, by practice. These results may present autonomy improvement of motor task executive centers (motor and visual) and cognitive control center (cognitive module) by practice, to process information more independently and more efficiently. Also, integration between motor-cognitive (θ), motor-visual (γ), and cognitive-visual (all frequencies) can be proposed as informative features to estimate practice time each individual trainee needs to learn RAS skills.

We also investigated whether long gap in practice would affect brain network dynamic architecture. Results showed that dynamic architecture of brain functioning may not be permanent and be affected by factors such as practice gap. Decrease of motor module recruitment (at α and β) and visual module recruitment (at β) with practice gap may point out that for a shorter RAS learning curve, trainees need to practice regularly and continuously. Hence, the deterioration of brain functioning changes as well as individual recovery rate should be considered in RAS learning curve. Since recordings in this study were limited to 6 sessions, we were not able to investigate the deterioration and recovery rate for individual subjects and also the effective factors on these variables. However, it seems change of integration and recruitment of motor and visual modules can provide useful information in this regard.

In addition to RAS learning architecture and its dynamic, we also explored change of brain functional network and cognitive features by outcome improvements, and the relationship between difficulty level, task complexity level, practice time, and cognitive and brain network features as well as FSRS metrics. Although we didn’t observe any significant relationship between cognitive features and FSRS metrics, detected significant correlation between strength and communication features and several FSRS metrics may suggest strength and communication as informative features to evaluate RAS outcome performance level. Results also showed that higher task complexity level is associated with higher number of erros, indicating lower performance level. Also, we found difficulty level was positively correlated with clutch usage, left tool grasp, number of errors, right tool grasp, right tool out of view, and tissue damage. This result indicates that higher difficulty level is associated with lower performance level. Results retrieved from correlation analysis showed that both task complexity level and difficulty level are effective variables on surgical outcome and performance level.

Our results highlighted several important opportunities to study RAS skill acquisition, cognitive neuroscience of learning, rehabilitation, and human-robot interaction challenges. Existing methods of objective RAS skill evaluation, in brain functioning analysis framework, are limited to cognitive features, extracted by using power spectral density analyses, which are unable to accurately depict detailed dynamic changes of brain functioning during learning. Use of brain functional network provides access to neurophysiological processes that open new insights into understanding learning and skill acquisition.

## Methods

### Experimental set up and data recording

**Fig 2:**
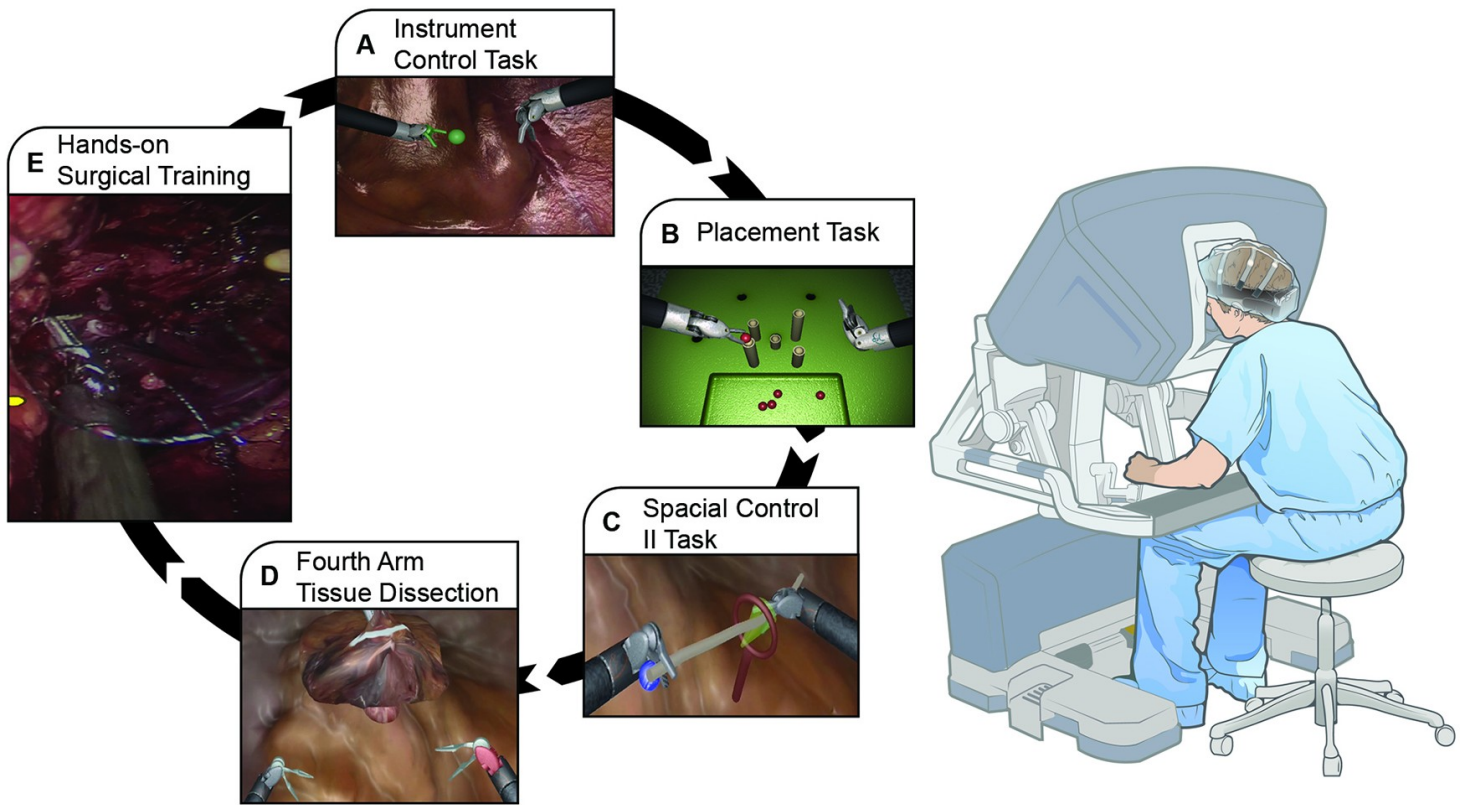
Illustration of experimental set up and schematic of RAS tasks included in this study to acquire skills related to human-machine interface. A 20-channel EEG headset was used to record trainee’s brain activity while performing tasks. Four surgical tasks were designed using Fundamental Skills of Robotic Surgery (FSRS) curriculum: (A) Instrument control task, (B) Placement Task, (C) Spatial control II task, (D) Fourth arm tissue dissection. (E) Hands-on surgical task performed by subjects on Robotic Surgery Simulator (RoSS).

We designed an experimental set up, where Electroencephalography (EEG) data from 27 subjects was recorded during six sessions throughout one year of practice, performing five RAS tasks (Fig 2). The study was conducted in accordance with relevant guidelines and regulations, and were approved by Roswell Park Cancer Institute Institutional Review Board (IRB: I-241913). Each subject provided written informed consent before participating.

#### Subjects

Twenty-seven medical students without any experience in robotic surgery and human-robot interaction. Participant characteristics were summarized in Table 5.

**Table 5:** Demographics.

Participant characteristics	Characteristics options	Number of participants
Age, years	<30	12
	30–45	15
Dominant Hand	Right	25
	Left	2
Gender	Male	17
	Female	10
Simulator Experience (and gaming)	No experience	27

#### Surgical tasks

Simulation based robotic curriculum—fundamental skills of robotic surgery (FSRS)—is a virtual reality-based curriculum that contains 4 modules (orientation, motor skills, basic surgical skills, and intermediate surgical skills) and 16 tasks [30]. Five surgical tasks, selected from FSRS curriculum focusing on human-machine interface skills, were included in this study:

1. Instrument Control Task (Fig 2A). This is task one of module one of FSRS and is designed for subject knowledge improvement. During this task subjects learn how to move the arms, which helps orient the user to the feel of the Robotic Surgery Simulator (RoSS) console [31].
2. Ball Placement Task (Fig 2B). This task involves subtasks 5 and 6 of module 2 of FSRS curriculum and is designed to teach psychomotor skills. The trainee is presented with five balls and five columns. Subjects pick up the balls from the tray using the robot simulator tools and place them on top of the columns. The task is completed when a ball is placed on top of every column [31]. Competence of the subjects for this task is evaluated using measures like the time taken to complete the task (Completion Time—CT), the number of times tool-tool collision occurs, and the number of times the camera is used and the clutch pedal is pressed [31].
3. Spatial Control II Task (threading string through a series of hoops) (Fig 2C). This task includes subtasks 7 and 8 of module 2 of FSRS curriculum and is designed to teach psychomotor skills. The subject passes a thread through a series of rings to hone his/her spatial awareness, instrument control and fine motor skills.
4. Fourth Arm Tissue Retraction (Fig 2D). This task is third task of module one of FSRS curriculum and is in the level of intermediate surgical skills [31]. This task combines the trainee’s previously acquired skills and requires coordinated control of the 4^*th*^ arm to retract tissue [31].
5. Hands-on Surgical Training (HoST) Anastomosis (Fig 2E). This task is designed to teach advanced RAS skills, and is completely different from the other four tasks. In HoST, a simulator has been developed using the hand movements of a master surgeon, and the trainee’s hands follow the master’s hand motion. It feels like a master surgeon holds the hands of the trainee and accompanies him/her throughout the performance. When the trainee’s hand movement is not correct, the program pushes his/her hands to the correct path.

FSRS scores were not available for cases that trainee could not finish task during maximum allowed time. In these cases, RoSS system automatically closed the task without reporting the FSRS scores (260 recordings out of 524). Number of recordings for each task and session were reported in Table 6.

**Table 6:** Number of total recordings and number of recordings with FSRS scores, while 27 RAS trainees performed 5 tasks during 6 sessions with various number of repeatitions.

Task	Session 1		Session 2		Session 3		Session 4		Session 5		Session 6	
	Total cases	With FSRS	Total cases	With FSRS	Total cases	With FSRS	Total cases	With FSRS	Total cases	With FSRS	Total cases	With FSRS
1	21	15	22	13	22	14	17	16	13	10	7	7
2	24	17	22	12	22	12	17	14	13	10	7	7
3	32	8	27	11	23	9	17	11	12	7	7	3
4	22	17	20	11	15	13	17	13	13	9	7	5
5	122	0	118	0	94	0	85	0	61	0	35	0

### Data

Twenty-seven subjects started learning robot-assisted surgery from the pre-novice level, and during one year of practice they learnt required skills to do the basic tasks of RAS on the simulator. EEG data recording was carried out at the initial session and followed up after one week, one month, three months, six months, and one year.

EEG data was recorded by placing the channels sensors of a 20-channel EEG headset on the Frontal (F; cognition and action; F3, Fz, F4, F7, F8 electrode channels), Prefrontal (PF; cognition; Fp1, Fp2 electrode channels), Central (C; action; C3, Cz, C4 electrode channels), Temporal (T; perception; T3, T4, T5, T6 electrode channels), Parietal (Pa; cognition; P3, Pz, P4, Poz electrode channels), and Occipital (O; perception; O1, O2 electrode channels) cortices (Fig 2). All surgical tasks were done on RoSS system.

#### EEG data preprocessing

The EEG data from the channels was filtered with a band-pass filter (0.5–128 Hz). Eye blinks, muscle activity, and environmental effects were considered as artifacts. Environmental artifacts were removed by applying a 60 Hz notch filter to EEG data [32, 33]. Muscle activity and eye movement were detected using wavelet transform and discriminant function analyses (DFA) as proposed by Berka et. al [33]. Linear discriminant function analysis was applied to raw data to decontaminate it from eye blinks. Wavelet coefficients from FzPOz and CzPOz channels were used to classify each signal data points into eye-blink or non-eye-blink categories [32]. The eye-blink data points were removed from signal.

### Brain functional network and community detection

A continuous wavelet transform with the ‘morlet’ wavelet method was used to extract the spontaneous phase of each channel signal for all recordings. The calculated phases of pair signals were used to find phase synchronized time points. These points were used to extract phase synchronization index for pairs of signals [34]. Calculating the phase difference (Δφ_xy_(t)) between two signals x(t) and y(t) by the following equation, the phase synchronized point was defined as time point (t) in which $\frac{\text{d}(\Delta \varphi (\text{t}))}{\text{dt}}=0$ [35].

$$\Delta \varphi_{\text{xy}}(\text{t})=|\varphi_{\text{x}}(\text{t})-\varphi_{\text{y}}(\text{t})|$$

Transferring the range of phase into boundary of *φ*_*x*_ ∈ [−*π*, *π*], the phase difference for all pairs of channels was normalized by using the range of phase difference ($\Delta \varphi_{\text{xy}}^{\text{max}}=2\pi$ and $\Delta \varphi_{\text{xy}}^{\text{min}}=0$) [35]. The synchronization index Γ_xy_(FB) was calculated through frequency band (FB) using [35, 36]

$$\Gamma_{\text{xy}}(\text{FB})=\frac{\sqrt{{\left[\sum_{\text{t}}\text{cos}(\Delta \varphi_{\text{x},\text{y}}^{\text{FB}}(\text{t}))\right]}^{2}+{\left[\sum_{\text{t}}\text{sin}(\Delta \varphi_{\text{x},\text{y}}^{\text{FB}}(\text{t}))\right]}^{2}}}{{\text{P}}_{\text{s}}}$$

where, P_s_ is the number of data-points in the time series. The calculated synchronization index for all pairs of channels forms the symmetric weighted adjacency matrix (Γ) [35]. The single-layer community detection algorithm was used to extract functional communities of recordings for all subjects through learning sessions. Communities were extracted through (4–60 Hz) with frequency bands of θ(4-8Hz), α(8-12Hz), β(12-35Hz), and γ(35-60Hz).

Single-layer community detection algorithm was used to detect communities of each adjacency matrix, which we defined these communities as functional states.

#### Single- layer community detection

We partitioned each adjacency matrix into communities (functional states) using modularity maximization criteria [37]. Modularity index (Q), as a quality function, was defined as [37].

$$\text{Q}=\sum_{\text{ij}}[\Gamma_{\text{ij}}-\gamma {\text{M}}_{\text{ij}}]\delta ({\text{g}}_{\text{i}},{\text{g}}_{\text{j}}),\;\;\;\;\;\;\;\delta ({\text{g}}_{\text{i}},{\text{g}}_{\text{j}})=\{\begin{array}{cc} 1 & {\text{g}}_{\text{i}}={\text{g}}_{\text{j}}\\ 0 & {\text{g}}_{\text{i}}\neq {\text{g}}_{\text{j}} \end{array}$$

where, channel ‘i’ is assigned to cluster g_i_, channel ‘j’ is assigned to cluster g_j_, and M_ij_ is the expected weight of the link connecting channels i and j. The coefficient γ>0 is the resolution parameter in the multiscale modularity-maximization algorithm. Maximization of Q is equivalent to partitioning the matrix into communities such that the total link weight inside the modules is as large as possible. The *generalized Louvain like “greedy” algorithm* and ‘Newman-Girvan (NG)’ null network were used for modularity maximization and community detection [37, 38]. Elements of matrix M are defined as $\frac{{\text{k}}_{\text{i}}{\text{k}}_{\text{j}}}{2\text{m}}$ for vertices i and j, where m is defined as $\text{m}=\frac{1}{2}\sum_{\text{i}}{\text{k}}_{\text{i}}$ and $\frac{{\text{k}}_{\text{i}}{\text{k}}_{\text{j}}}{2\text{m}}$ is the degree of the vertices. Expression $\sum_{\text{ij}}(\Gamma_{\text{ij}}-\frac{{\text{k}}_{\text{i}}{\text{k}}_{\text{j}}}{2\text{m}})$ is called the modularity matrix.

To find the optimum consistent modularity and resolution parameter for each adjacency matrix Γ, a range of γ was considered and ‘Q’ was calculated at each γ (using generalized Louvain algorithm).

**Fig 3:**
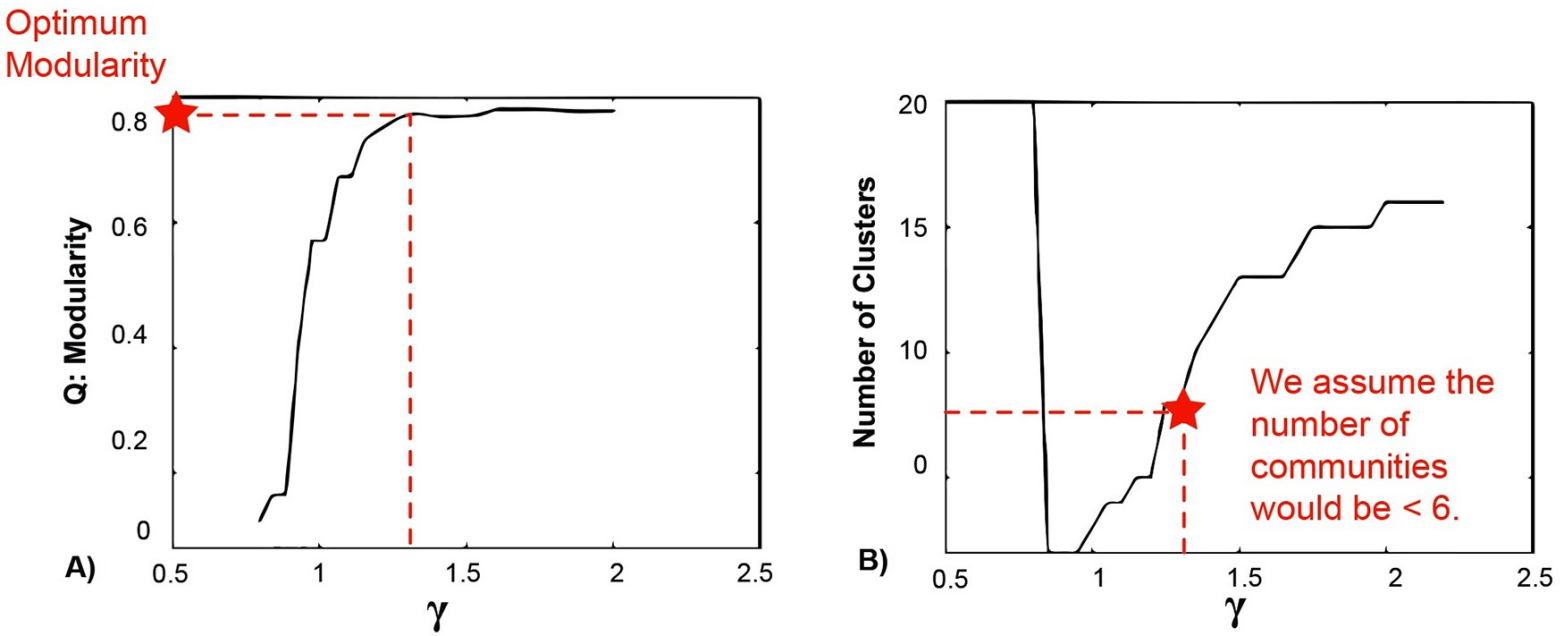
Illustration of selecting optimum modularity value.

Finally, the resolution limit that resulted in maximum modularity and acceptable number of communities (≤ 6 in this study) was selected as the optimum resolution limit for each matrix (Fig 3). We selected the number of communities ≤ 6 because the network in this study was small with 20 nodes and we considered 6 brain predefined cognitive systems. Considering more than 6 communities resulted in several singular communities, which do not convey meaningful information toward our purpose.

### Module-allegiance matrix, brain functional network measurements

**Fig 4:**
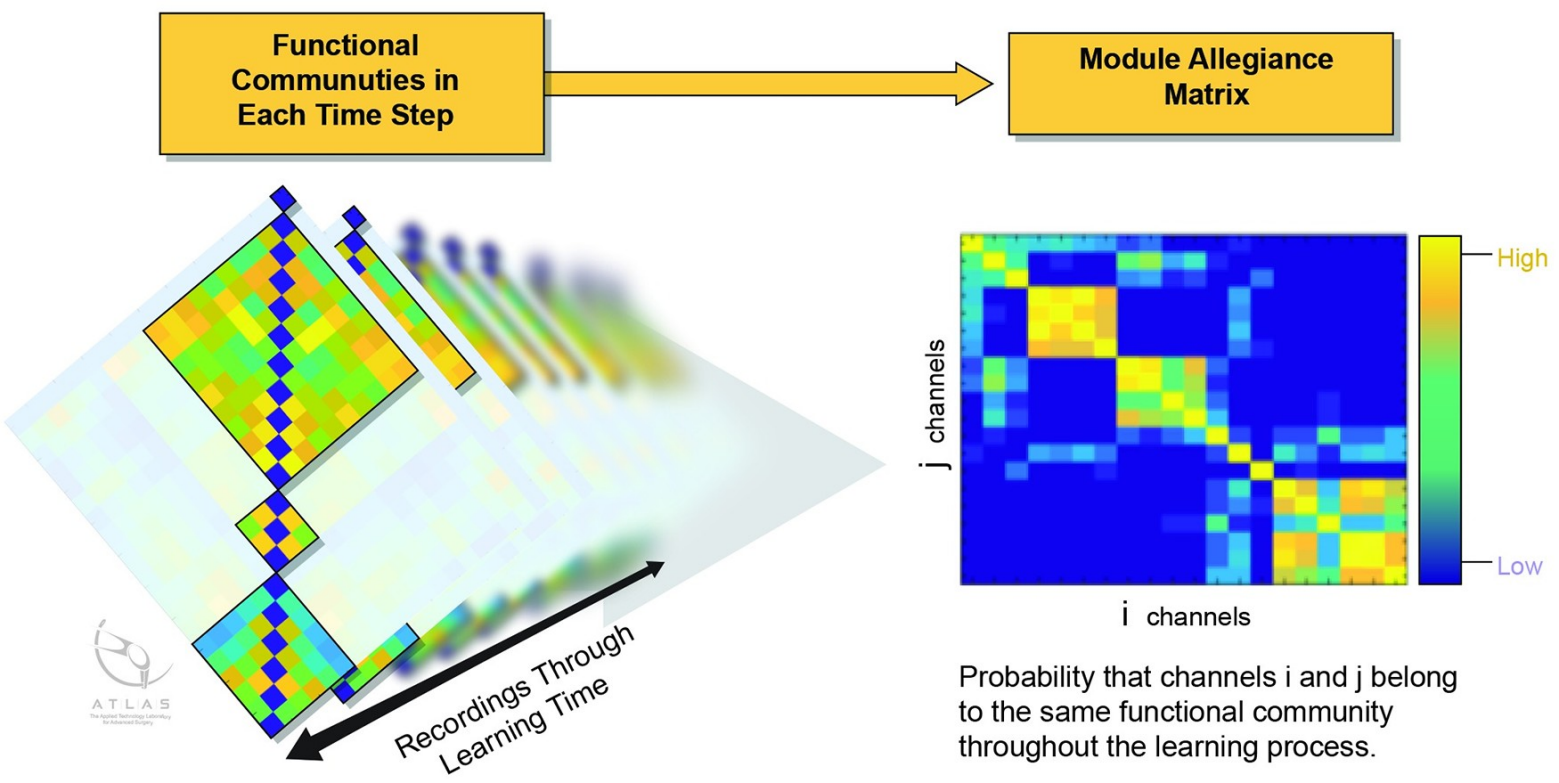
Schematic of module allegiance matrix extraction process. Functional connectivity values were used in adjacency matrix, which were then used in multilayer community detection algorithm. Extracted functional communities for all recordings were used to extract MAM.

Using the functional community each channel was assigned to, the Module Allegiance Matrix (MAM) was derived. Element (i,j) in the MAM represents the probability that nodes ‘i’ and ‘j’ belong to the same community (Fig 4).

**Fig 5:**
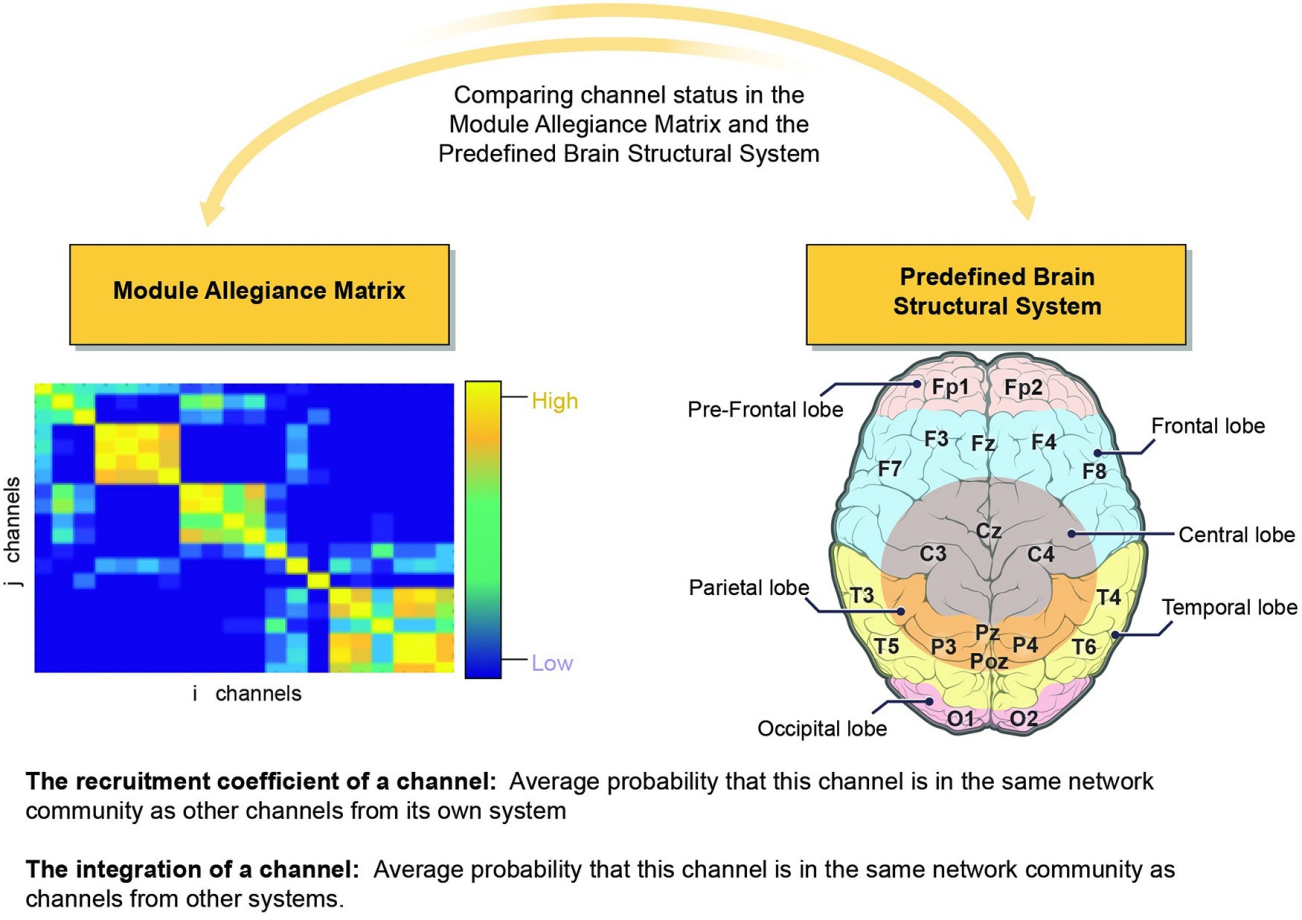
Comparison between the structural community of the channels and the MAM was used to extract the integration coefficient, and recruitment coefficient.

Comparison between module allegiance matrix and predefined brain structural system was used to extract brain state reconfiguration measurements. Considering the structural connectivity of considered channels (represented in Fig 5), the integration and recruitment coefficients were calculated.

#### Features

Different categories of features were measured (NASA-TLX metrics and FSRS metrics) and calculated (cognitive and functional network features) to find the relationship between subjective assessment metrics (NASA-TLX), real outcome measures (FSRS metrics and completion time (CT)) and analytical features retrieved from brain activity (measured by EEG).

#### Subjective metrics

The trainee evaluated each procedure by giving scores to NASA-TLX indices, with a scale of 1 to 20. This subjective skill evaluation survey has six indices: Mental Demand, Physical Demand, Temporal Demand, Performance, Effort, and Frustration [39, 40].

- Mental Demand (MD): Evaluates the level of mental/perceptual activity demanded to complete the task.
- Physical Demand(PD): Level of physical activity required to complete the task.
- Temporal Demand(TD): Level of time pressure the subject feels during completing the task.
- Performance Score (PS): Quality level of outcome and the level of satisfaction of doing the task (1 means highest quality and 20 means lowest quality).
- Effort (E): Evaluates how hard (mentally and physically) should the subject work to complete the task.
- Frustration: Level of negative (compared to positive) psychological emotions the subject feels while completing the task.

**Task difficulty**: We used scores given to these metrics to extract task difficulty (D) level as

D=MD+PD+TD+E

We did not use performance scores given by trainee because trainee does not have enough expertise to be able to evaluate outcome of performed task.

**Task complexity**: FSRS curriculum modules were used to determine the complexity level of tasks in Table 7, with 1 being simplest and 5 most complicated task.

**Table 7:** Description of tasks designed in this study, and the reason for the associated skill necessity in RAS.

Task name	Instument control	Ball placement	Spatial control II	Fourth arm tissue dissection	Hands-on surgical training
Task ID	(1)	(2)	(3)	(4)	(5)
Complexity level	2	4	5	3	1
Skill	orientation and control of position	Hand-eye-tool coordination & depth perception & foot control	Hand-eye-tool coordination & depth perception & foot control	orientation and control of position	Motor skills
Reason	Lack of tactile feedback in RAS requires development of this skill	Remoteness and lack of tactile feedback requires development of this skill	Remoteness and lack of tactile feedback requires development of this skill	Lack of tactile feedback in RAS requires development of this skill	Learn how to have fine hand movements

#### FSRS metrics

RoSS scores given by da Vinci simulator technology were used in this study for outcome evaluation. These metrics include: clutch usage, left tool out of view, number of errors, right tool out of view, tissue damage, and tool-tool collision. These metrics were used in correlation analysis, to investigate the relationship between brain state measurements and RoSS scores. These scores were also used to estimate performance level of doing each task by subjects. Performance level was calculated as the average of scores given to all 8 FSRS metrics as

$$\text{Performance}=100-\frac{1}{8}\sum_{\text{i}=1}^{8}\text{FSRS}(\text{i})$$

#### Completion time

By synchronizing the recorded EEG and the associated video of the task, completion time was defined as the total time a trainee was performing a task. The completion time can be defined using the number of total data points in signal (N) and the data recording sampling frequency (f_s_) as

$$\text{CT}=\frac{\text{N}}{{\text{f}}_{\text{s}}}$$

#### Cognitive features- power spectral density

**Aiming period activity**: Brain activity during short and long term decision making affects the performance while operating a procedure [41]. The whole of hand movement is planned and figured out during long-term decision making, which happens during the Aiming Period (AP). This period has revealed important effects on performance and expertise level in different tasks [41–43]. The brain activity during this period is Aiming Period Activity (APA), which is the power reduction in spatially enhanced α(8–12 Hz) and β(13–30 Hz) bands during movement [43]. It has been shown that the APA depends on the expertise level of the subject [41].

**Mental Workload**: Mental workload (MW) is related to the engaged memory capacity while performing a procedure of interest [44]. The main assumption in MW interpretation is that each person has a relatively fixed cognitive capacity [32]. Commonly, MW refers to the portion of a person’s total mental capacity which is loaded [45]. Mental workload was estimated using the PSD of signals at the C3-C4, Cz-POz, F3-Cz, F3-C4, Fz-C3, and Fz-POz channels [32].

To calculate MW, we used the framework developed by the B-Alert EEG series from Advanced Brain Monitoring (ABM) company, which has been frequently validated in different studies [32, 33, 46]. Briefly, this framework calculates a baseline value of the absolute and relative power spectral variables from the C3-C4, Cz-PO, F3-Cz, Fz-C3, and Fz-PO channels during mental arithmetic, grid location, and digit-span baseline tasks. These baselines have been recorded from 80 healthy subjects, and are available from ABM software. A two-class quadratic logistic discriminant function analysis (DFA) [32] was used to extract the probability of presenting a high mental workload. The quadratic logistic DFA was established once for one mentor based on baseline data collected before surgeries.

#### Engagement

Engagement reflects the spatial recruitment of the brain regions in processing tasks associated with decision making. These tasks include, but are not limited to, information gathering, visual scanning, audio processing, and attention concentration on one aspect of the environment while ignoring other distractions [32, 33]. As with the calculation of MW, we used the framework developed by the B-Alert EEG series from Advanced Brain Monitoring (ABM) company. However, in this case, the baselines were drawn from 5 minutes of three different tasks (3-choice vigilance task, eyes open, and eyes closed).These baselines were recorded from one mentor at the beginning of the whole research study. Here, the absolute and relative PSD values of the Fz-POz and Cz-POz channels were used in a four-class quadratic logistic discriminant function analysis (DFA) which returned an estimation of the engagement level [32, 33]. The range of this estimation for 1 second epoch is between 0–1 with 0.1 (sleep onset), 0.3 (distraction), 0.6 (low engagement; LE), and 0.9 (high engagement; HE).

#### Asymmetry index

Negative feelings such as surprise, frustration, fear, and concern have opposing effects on the activity of the right and left lobes of the frontal cortex [47]. For each recording, Asymmetry Index (AI) was defined as the difference between the power spectral density decrease in the left and right frontal lobes in the alpha frequency band, normalized as [47, 48]

$$\begin{array}{l} \text{AI}=\frac{\text{L}-\text{R}}{\text{L}+\text{R}}\\ \text{L}={\text{PSD}}_{\text{max}}^{\text{F}3}(\alpha )-{\text{PSD}}_{\text{min}}^{\text{F}3}(\alpha )+{\text{PSD}}_{\text{max}}^{\text{F}7}(\alpha )-{\text{PSD}}_{\text{min}}^{\text{F}7}(\alpha )\\ \text{R}={\text{PSD}}_{\text{max}}^{\text{F}4}(\alpha )-{\text{PSD}}_{\text{min}}^{\text{F}4}(\alpha )+{\text{PSD}}_{\text{max}}^{\text{F}8}(\alpha )-{\text{PSD}}_{\text{min}}^{\text{F}8}(\alpha ) \end{array}$$

The AI was calculated as the average value over the following pairs of electrodes: (F3 and F4), and (F7 and F8). During negative feelings, the right frontal lobe shows more intense activity (associated with lowerα power [48]) compared to the left lobe [48] (α power is inversely related to activation [47]).

#### Brain functional network features

**Strength**. The strength of a cognitive system was defined as the average functional connectivity of electrodes within the system. Adjacency matrices were used to calculate this feature.

**Communication**. Communication, C, between two cognitive systems k_**1**_ and k_**2**_ can be defined as the average functional connectivity in electrode pairs where one electrode lies within the first system and the second electrode lies within the second system [49]. Adjacency matrices (Γ) were used to calculate this feature as

$${\text{C}}_{{\text{k}}_{1},{\text{k}}_{2}}=\frac{\sum_{\text{i}\in {\text{k}}_{1}\text{j}\in {\text{k}}_{2}}\Gamma_{\text{ij}}}{\left(|{\text{S}}_{{\text{k}}_{1}}||{\text{S}}_{{\text{k}}_{2}}|\right)}$$

where, |S_k_| is the number of nodes in the cognitive system k, k = 1…6, and k_1_ ≠ k_2_. Note that the strength of each cognitive system can be calculated by letting k_1_ = k_2_.

**Integration coefficient**. Integration of each area measures the average probability that this area is in the same network community as areas from other systems. Module Allegiance Matrices were used to calculate integration feature.

**Recruitment coefficient**. The recruitment coefficient for each area of the network corresponds to the average probability that this area is in the same network community as other areas from its own system. Module Allegiance Matrices were used to calculate recruitment feature.

All features used in this study were summarized in Table 8.

**Table 8:** Summary of all features used in this study. Four feature categories were considered: Subjective assessment metric, tool-based metrics, cognition, and brain functional network features.

Feature	Description	Category	Main extraction method
**Difficulty level (D)**	Extracted by using NASA-TLX features, evaluated by the trainee D = MD + PD + TD + E	Subjective assessment	Subjective assessment
**Complexity level**	Indicates the complexity of task based on assessment of expert surgeons considered in FSRS curriculum. This feature is independent from trainee performance and outcome and is based on the properties of the task.	FSRS curriculum	FSRS curriculum
**Practice time**	All subjects started learning without any experience of work with simulator, gaming, and robotic surgery experience. However, during learning they practice on simulator and their experience increases from session to session. We measured practice time of each subject from recording to next recording and considered that (seconds of practice) as practice time.	Direct measurement	Direct measurement
**Practice gap**	Gap between practice sessions	Direct measurement	Direct measurement
**Clutch usage**	Indicates effectiveness and the level of skill in which the task is performed (economy of motion) [50]	Tool besed metric (FSRS)	Real measurement by RoSS
**Left tool out of view**	Indicates awareness of operative environment in which tasks are Performed [50]	Tool besed metric (FSRS)	Real measurement by RoSS
**Right tool out of view**	Indicates awareness of operative environment in which tasks are performed [50]	Tool besed metric (FSRS)	Real measurement by RoSS
**Number of errors**	Indicates effectiveness and the level of skill in which the task is performed (economy of motion) [50]	Tool besed metric (FSRS)	Real measurement by RoSS
**Tissue damage**	Collisions causing damage to the underlying tissue [31]. Indicates awareness of operative environment in which tasks are performed [50]	Tool besed metric (FSRS)	Real measurement by RoSS
**Tool-Tool collision**	Indicates awareness of operative environment in which tasks are performed [50]	Tool besed metric (FSRS)	Real measurement by RoSS
**Performance Level**	Performance level was calculated as: $100-\frac{1}{8}\sum_{\text{i}=1}^{8}\text{FSRS}(\text{i})$	Tool besed metric (FSRS)	Real measurement by RoSS
**Completion time (CT)**	Indicates total time task is performed	Direct measurement	
**Aiming period activity (APA)**	Indicates brain activity level during aiming period	Cognition	Power Spectral Density (PSD) analysis
**Mental Workload (MW)**	Indicates the level of engaged memory capacity while performing task	Cognition	Power Spectral Density (PSD) analysis
**Engagement (E)**	Reflects the spatial cooperation of the brain regions in processing tasks	Cognition	Power Spectral Density (PSD) analysis
**Strength**	Indicates the level of total functional connectivity within channels in a specific cognitive system	Brain functional network	Pairwise phase synchronization
**Communication**	Indicates the level of total functional connectivity between channels from different cognitive systems	Brain functional network	Pairwise phase synchronization
**Integration**	Average probability that a brain area is in the same network community as areas from other cognitive systems	Dynamic architecture feature	Network community detection
**Recruitment**	Average probability that a brain area is in the same network community as other areas from its own cognitive system	Dynamic architecture feature	Network community detection

## Conclusion

Current analysis frameworks are unable to describe necessary dynamic changes of brain areas to make performance of motor cognitive skills autonomous, because of statistical and mathematical limitations. In this study we used dynamic network neuroscience methods to investigate dynamic reconfiguration of brain modules during RAS skill learning, effective factors on dynamic architecture of learning, and address several questions related to RAS skill acquisition. Results also suggest integration, recruitment, strength, and communication features to be used for objective performance evaluation.

## Supporting information

S1 File ‘Module allegiance matrix’ data through different sessions and frequency bands of θ, α, βγ. Description file explains data.
(ZIP)

S2 File Recruitment, integration, total practice time for subjects up to each session (second), gap between practice sessions, and performance level data for six sessions. Description file explains data.
(ZIP)

S3 File Features for 260 recordings used in this study. Data required for extraction of Tables 3 and 4, and the description file. Description file explains data.
(ZIP)
